# Se improves GPX4 expression and SOD activity to alleviate heat-stress-induced ferroptosis-like death in goat mammary epithelial cells

**DOI:** 10.1080/19768354.2021.1988704

**Published:** 2021-10-17

**Authors:** Lu Liu, Manjiang Wang, Ning Gong, Peng Tian, Hongxia Deng

**Affiliations:** a College of Chemistry & Pharmacy, Northwest Agricultural & Forestry University, Yangling, People’s Republic of China; b Fuping County Animal Epidemic Prevention Control Center, Xianyang, People’s Republic of China

**Keywords:** Ferroptosis, selenium, heat stress, GPX4, SOD

## Abstract

Selenium (Se) is a vital element of life, which has an important impact on the growth, development, production performance and stress-tolerance of animals. However, it is not entirely clear that how exactly Se works during these processes. Herein, we investigate the role of Se in regulating the functions of goat mammary epithelial cells (GMECs) under heat-stress condition. We found that heat stress caused ferroptosis-like death in GMECs, manifested by a robust increase in iron ion concentration, reactive oxygen species (ROS) and cell death ratio, and a decrease in the activity of superoxide dismutase (SOD) and expression level of glutathione peroxidases 4 (GPX4). Se incubation had no obvious effect on GMEC viability, but alleviated heat-stress-induced ferroptosis-like cell death and improved GPX4 expression and SOD activity in a dose-dependent manner. Also, we found that overexpression of GPX4 could improve the activity of SOD. And Se incubation inhibited activation of mTOR signaling in heat-stress-induced GMECs, which could be eliminated by the mTOR activator MHY1485, and treatment with mTOR inhibitor AY-22989 had the same effect as Se. In conclusion, Se improves GPX4 expression and SOD activity and inhibits the activation of mTOR to alleviate heat-stress-induced ferroptosis-like death in GMECs, which may be a protective agent for heat stress in goats.

## Introduction

Ferroptosis is a recently recognized type of non-apoptotic cell death and its main signs are the accumulation of ROS in the cytoplasm and mitochondria and the damage of mitochondrial structure and function (Xie et al. 2016). Initially, a new compound erastin is identified as the main cause of ferroptosis caused by the induction of Ras proto-oncogene expression in human foreskin fibroblasts (BJeLR) (Dolma et al. 2003). Later, another study showed that Ras-selective lethal small molecule (RSL)-3 and RSL5 could induce ferroptosis of human foreskin fibroblasts (BJeLR) (Yang and Stockwell 2008). Currently, the causes of ferroptosis-like death are still defined as various small molecules that induce mutations in Ras family genes (Dixon et al. 2012). Ferroptosis is detected in oxidative damage to various organs, including kidney (Eleftheriadis et al. 2019), lung (Yoshida et al. 2019), intestine (Qi et al. 2020), testis (Jia et al. 2018), etc., however, there is no related report on mammary gland. The major cell type in mammary gland is mammary epithelial cells (MECs), which plays an important role for milk synthesis (Saipin et al. 2018). A previous report stated that during the lactation period of goats, exposure to heat stress tend to reduce mammary plasma flow relative to thermal neutrality, thereby reducing lactation (Salama et al. 2020). And it is reported that heat stress could significantly increase the expression of related molecules in goat mammary glands and induce ferroptosis (Gnanapradeepan et al. 2018; Hooper et al. 2020). Therefore, we speculated that heat stress may cause ferroptosis of goat mammary epithelial cells and affect lactation, but the specific molecular mechanism in this process is still unclear.

At present, a large number of studies reported that glutathione peroxidase 4 (GPX4) is the core of controlling cell ferroptosis-like death (Gaschler et al. 2018; Tobias et al. 2018). This is due to the fact that only GPX4, one of the five known glutathione peroxidase (GPX) subtypes, is confirmed to be the enzyme that plays a major role in the lipid peroxidation process (Zemolin et al. 2012). In this process, GPX4 converts potentially toxic lipid hydroperoxides (L-OOH) to non-toxic lipid alcohols (L-OH), thereby eliminating the accumulation of ROS in the body and alleviating ferroptosis-like death (Stockwell et al. 2017). The elimination of ROS is coordinated by a variety of enzymes. Among them, the superoxide dismutase (SOD) and glutathione peroxidase systems are confirmed to play a role in this process. After ROS production, ROS is rapidly converted by SODs to H_2_O_2_. Newly formed H_2_O_2_ is converted to H_2_O + O_2_ by GPX through coupling with reduced glutathione (GSH) and its subsequent conversion to oxidized glutathione (Trachootham et al. 2009; Peng et al. 2019). Moreover, it is reported that GPX4 is a selenium (Se)-containing enzyme (Angeli and Conrad 2018; Ingold et al. 2018). And studies reported that adding selenium to the diet could promote the expression of GPX4 and the activity of SOD to improve the antioxidant capacity of animals (Chen et al. 2017; Khan et al. 2018; Mengistu et al. 2021), but how selenium plays its role in ferroptosis-like death is unclear.

mTOR is gradually introduced into people's vision with emerging investigations proposed that the abnormal regulation of mTOR signaling pathway is closely related to cell proliferation, apoptosis and autophagy (Han et al. 2020). A study found that the overexpression of mTOR suppressed Fe (III)-citrate, erastin, and RSL3-induced cell ferroptosis-like death, whereas mTOR deletion exaggerated cell death in these conditions (Baba et al. 2018). While, a study also found that the deactivation of mammalian target of rapamycin (mTOR) could indirectly lead to the increase of SOD expression, which ultimately lead to ROS reduction and inhibition of ferroptosis (Li et al. 2019). However, it is reported that the activation of mTOR signal in acute myeloid leukemia (AML) cells could promote the accumulation of ROS, and ultimately lead to cell iron death (Du et al. 2019). A study found that when the mTOR signaling pathway is inhibited in the human trophoblast cell line HTR8/SV neo and the pig trophoblast cell line pTr2, the level of GPX4 is increased, and autophagy and iron disease are decreased (Han et al. 2020). It can be seen that the influence of mTOR signaling pathway on ferroptosis is still controversial.

Our research aims to explore whether selenium incubation could alleviate the ferroptosis-like death of goat mammary epithelial cells induced by heat stress and associated molecular pathways that selenium involved, so as to provide a new idea for increasing goat milk production under heat stress.

## Materials and methods

### Measurement of selenium concentration in tissues

This experiment used 1-year-old common healthy adult goats without pregnant (Xi’an Experimental Animal Center). The experiment cycle was 4 weeks. All procedures involving experimental animals in this study were approved by the Ethics Committee of Northwest Agricultural & Forestry University. The goats in the control group were raised at room temperature in 20°C ± 2°C, and the heat stress group was placed at 40°C ± 2°C greenhouse culture, normal feeding and watering, each group had 6 goats.

The goats were slaughtered and the tissues of each corresponding part were used for the test. Selenium concentration in tissues was analyzed by dilution-hydride generation-inductively coupled plasma-mass spectrometry (ID-HG-ICP-MS), as reported (Kleckner et al. 2017). Briefly, 10–15 mg of freeze-dried, homogenized tissue samples were weighed, and an appropriate amount of enriched 82Se isotope spike standard solution and 600 μL of 16 NHNO_3_ were added to 6 mL PFA vial (Savillex, 10,321 West 70th Street Eden Prairie, MN 55344-3446 USA part number 200-006-20), and the PFA vial was sealed and heated in a benchtop autoclave at 126°C/20 psi for 3 h. After cooling in the dark, 200 μL of 30% H_2_O_2_ were added to the PFA vial to oxidize. After full reaction, electric furnace was used to heat it to dry. An equal volume of 2% HCl was added to reduce the selenium in the sample to selenite. The Se concentration was analyzed by ICP-MS combined with a Flow Injection System FIAS 400 (Perkin-Elmer SCIEX, Shelton, Connecticut, USA) through Elan DRC II (Perkin-Elmer SCIEX, Shelton, Connecticut, USA) for hydride generation.

### Isolation and in vitro culture of goat mammary epithelial cells (GMECs)

GMECs were isolated from 1-year-old Saanen dairy goat (The 3rd farm of Northwest Agricultural & Forestry University) at peak lactation (15 days postpartum) as previously described (Wang et al. 2010). The isolated GMECs were cultured in DMEM medium (Invitrogen Corp., USA) containing 5 mg/mL insulin (Invitrogen Corp, USA), 0.25 mmol/L hydrocortisone, 10 ng/mL epidermal growth factor 1 (EGF-1, Gibco), 100 U/mL penicillin and 100 μg/mL streptomycin (Invitrogen Corp., USA), and 10% FBS at 37°C in a humidified atmosphere with 5% CO_2_ for subsequent experiments.

### Cell culture and transient heat treatment

Exponentially growing GMECs after 12 h of passage were seeded in culture dishes and treated with heat stress at 48°C for 10 min in a temperature-controlled water bath, as reported (Obara et al. 2008; Dore et al. 2014). Then, the culture medium was changed and cells were cultured under normal conditions. The cell cultures were terminated at the indicated time points for further analysis.

### Cell transfection and treatment

GPX4 siRNA and negative control siRNA were synthesized by Shanghai Gene Pharma Co., Ltd (Shanghai, China). GPX4 overexpression vector (pcDNA3.1-GPX4) and pcDNA3.1 empty vector used in this experiment were purchased from Qingke Biotechnology Co., Ltd. (Beijing, China). The day before transfection, we seeded cells at the density of 2×10^5^ cells/mL. After reaching 70% confluence, GMECs were transfected with the vectors or siRNAs by using Lipofectamine 2000 Transfection Reagent (Invitrogen) according to the manufacturer's instructions.

### Cell viability assay

The treated GMECs were seeded in a 96-well plate (3000 cells/well) and cultured for 24 h. After reaching 70% confluence, the cells were cultured in DMEM medium without FBS and incubated with different concentrations (1, 5, 10, and 20 nM) of selenium for 72 h. After that, cell viability was detected by using MTT assay. In brief, after the cells were cultured for the specified time, MTT reagent (20 μL, 5 mg/mL) was added to each well. After 4 h, the MTT-containing medium was removed, and DMSO (150 µL) was added to each well, then the absorbance of each well at 490 nm was measured with a microplate reader (Bio-Rad, Sunnyvale, CA, USA). viability rate (%) = (OD _sample_−OD _blank_) / (OD _control_−OD _blank_) ×100.

### Determination of ROS content

In this experiment, the 2’,7'-dichlorofluorescein diacetate (DCFH-DA) molecular probe (MedChemExpress, New Jersey, USA) was used to determine the intracellular ROS content. Cells were cultured in a 96-well plate till a specified time, then DCFH-DA (10 mM) was added to each well and incubated for 30 min at room temperature. After incubation, the fluorescence of DCFH-DA was measured at an excitation wavelength of 485 nm and an emission wavelength of 535 nm by using a fluorescent plate reader (Beckman, USA). The final content of ROS was expressed as relative fluorescence units.

### Iron content detection

The iron content in the treated GMECs was measured by using a sensitive iron colorimetric detector, terrocene S (LMWI). The specific steps were based on the manufacturer’s instructions.

### Measurement of SOD activity

We measured the SOD activity in GMECs by using the SOD determination kit (Nanjing Jiancheng Institute of Biological Engineering, Nanjing, China, A001-3-2) through a spectrophotometer and operated according to instructions. Briefly, SOD could inhibit the superoxide anion free radicals generated in the reaction system and oxidize hydroxylamine to generate nitrite. Colorimetric determination of nitrite in sample tubes and standard tubes the amount is defined according to enzyme activity: When the inhibition rate of SOD on nitrite reaches 50% per mg of tissue protein in 1 mL reaction solution, the corresponding amount of SOD is one SOD activity unit (U). The SOD activity can be calculated by the following formula (U/mg, the number of milligrams of protein): SOD activity in cells = {(standard tube absorbance-measuring tube absorbance)/standard tube absorbance/50%× (total volume of reaction system/sampling volume)}/ Protein content.

### Measurement of mTOR activity

The activity of mTOR in the GMECs was quantified by colorimetry according to the standard protocol in the mTOR kinase activity assay kit (Merck, Darmstadt, Germany, CBA055).

### Real-time PCR for genes expression

Total RNA was extracted from cells with TRIzol reagent (Thermo Fisher Scientific, Inc), and RNA was reverse-transcribed into cDNA by using a high-capacity cDNA reverse transcription kit (Applied Biosystems, Carlsbad, CA, USA) according to the instructions. RNA abundances were detected by using a final volume of 25 µL reaction system containing the Power SYBR Green PCR Master Mix (Applied Biosystems) in the Applied Biosystems 7500 Real-Time PCR System (Applied Biosystems, Carlsbad, CA, USA). The reaction system was incubated under the following conditions: 95°C for 1 min, followed by denaturation at 95°C for 15 s, annealing at 60°C for 25 s and extension at 72°C for 15 s for 35 cycles. β-tubulin was used as an endogenous control. Relative gene expression was quantified with the 2^-ΔΔCt^ method. The primers used in this research were: GPX4 forward primer 5'-TAC TGC AAC AGC TCC GAG TTC-3’, reverse primer 5'-GGT GCC AAA GAA AGA AAG TCC-3’; β-tubulin forward primer 5'-GAA TGG GCA CTC TCC TTA TC-3’, reverse primer 5'-GGA CCA TGT TGA CTG CCA AC-3’.

### Western blotting analysis

Cells were harvested after treatment and lysed with a lysis buffer (Sigma-Aldrich, Merck KGaA, Darmstadt, Germany) containing a protease inhibitor cocktail. The protein concentration was determined with a BCA protein assay kit (Beyotime Biotechnology, Shanghai, China) following the manufacturer’s instructions. Protein lysates were loaded onto 10% sodium dodecyl sulfate polyacrylamide gel for separation by electro-phoresis and then transferred onto a poly-vinylidene fluoride membrane (Millipore, Boston, MA). The membrane was immersed in 5% skim milk at 4°C overnight to block non-specific binding sites. Thereafter, the membrane was incubated with primary antibodies against GPX4 (1:1000, Abcam, Cambridge, MA, USA, ab125066), phosphorylated mTOR (p-mTOR, 1:1000, Abcam, ab109268), total mTOR (t-mTOR, 1:10000, Abcam, ab134903), p-S6K1 (1:500, Abcam, ab131436), t-S6K1 (1:5000, Abcam, ab32529), p-4EBP1 (1:1000, Abcam, ab75767), t-4EBP1 (1:1000, Abcam, ab32130) and β-tubulin (1:5000, Abcam, ab7291) at room temperature for 2 h. After washing with Tris-buffered saline with Tween 20 (TBST), the membrane was probed with horseradish peroxidase (HRP)-conjugated secondary antibody (1:2000, Jackson ImmunoResearch) at room temperature for 1 h. After elution, the expression levels of the proteins were analyzed via chemiluminescence and quantified by using ImageJ Software.

### Statistical analysis

Each measurement was obtained from at least triple experiments, and data were expressed as means ± standard error of mean (SEM). Data analysis was performed with SPSS22.0 (SPSS Inc. Chicago, Illinois, USA). Student's t-test was used to estimate the significance of differences between two unpaired samples, and ANOVA was used to compare differences among groups. Statistical significance was defined as *P* < 0.05.

## Results

### Selenium content in goat mammary gland tissues is significantly reduced under heat stress

**Figure 1.**
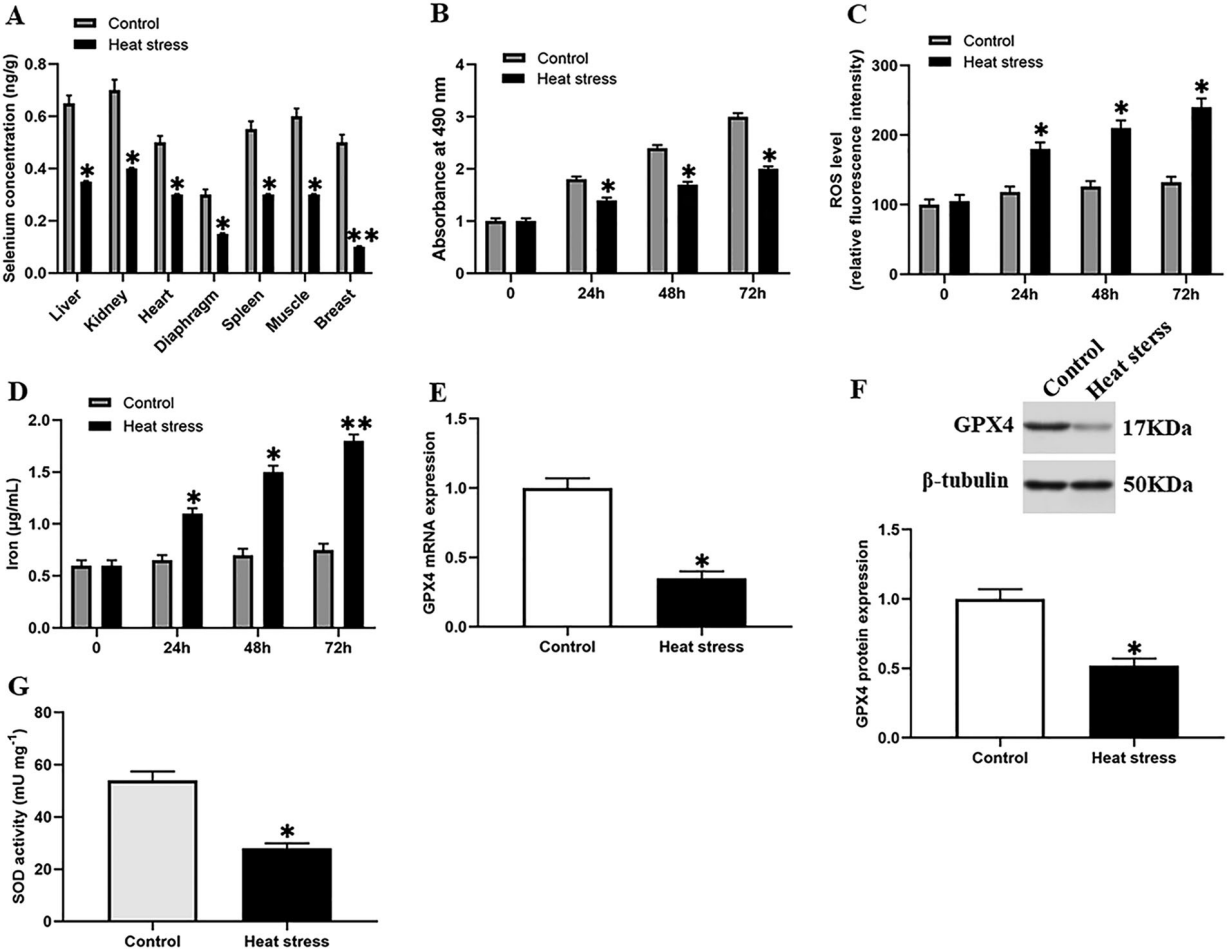
Heat stress induced ferroptosis-like death in GMECs. One-year-old normal healthy adult goats in experimental group or control group were raised in an environment of 40°C ± 2°C or 20°C ± 2°C for 4 weeks and then slaughtered, (A) each tissue was taken and the selenium content was determined using the ID-HG-ICP-MS method. The DMECs isolated from 1-year-old Saanen dairy goats at the peak lactation (15 days postpartum) were used for heat stress treatment, then the cell viability, iron ion concentration and ROS level were measured to determine the extent of ferroptosis: (B) MTT assay was used to detect the cell viability. (C) Determination of ROS content. (D) Determination of iron ion concentration by spectrophotometer. (E) RT-PCR was used to detect the expression of GPX4 nucleic acid. (F) Western blotting was used to detect the expression of GPX4 protein. (G) SOD determination kit was used to determine the activity of SOD. *P < 0.05 versus control, **P < 0.01 versus control, ***P < 0.001 versus control, n=6.

To explore the effect of heat stress on the selenium content in goat tissues, we divided goats of the same age and sex into two groups. The goats in the negative control group were raised at room temperature (20°C ± 2°C), and the goats in the heat stress group were raised in a greenhouse at 40°C ± 2°C for 4 weeks. We found that compared with the control group, the selenium content in goat organs under heat stress was significantly reduced (Figure 1(A)), and the selenium content in the breast tissues decreased the most, suggesting that heat stress has the greatest impact on goat breast tissues.

### Heat stress induces ferroptosis-like death in goat mammary epithelial cells

We performed heat stress treatment on GMECs and observed them for 72 h. During this period, cell viability, ROS level, and iron content were measured every 24 h. We found that compared with the control group, the cell viability of the heat stress group was significantly decreased (Figure 1(B)). On the contrary, the ROS accumulation and iron ion content of the heat stress group were increased significantly after 24 h, and reached a higher level at 72 h (Figure 1(C,D)). Our data showed that the heat stress may cause the ferroptosis-like death of cells. At the same time, we also found that the expression level of GPX4 and SOD activity were significantly decreased under heat stress (Figure 1(E,–G)).

### Selenium incubation reduces heat stress-induced ferroptosis-like death of GMECs

**Figure 2.**
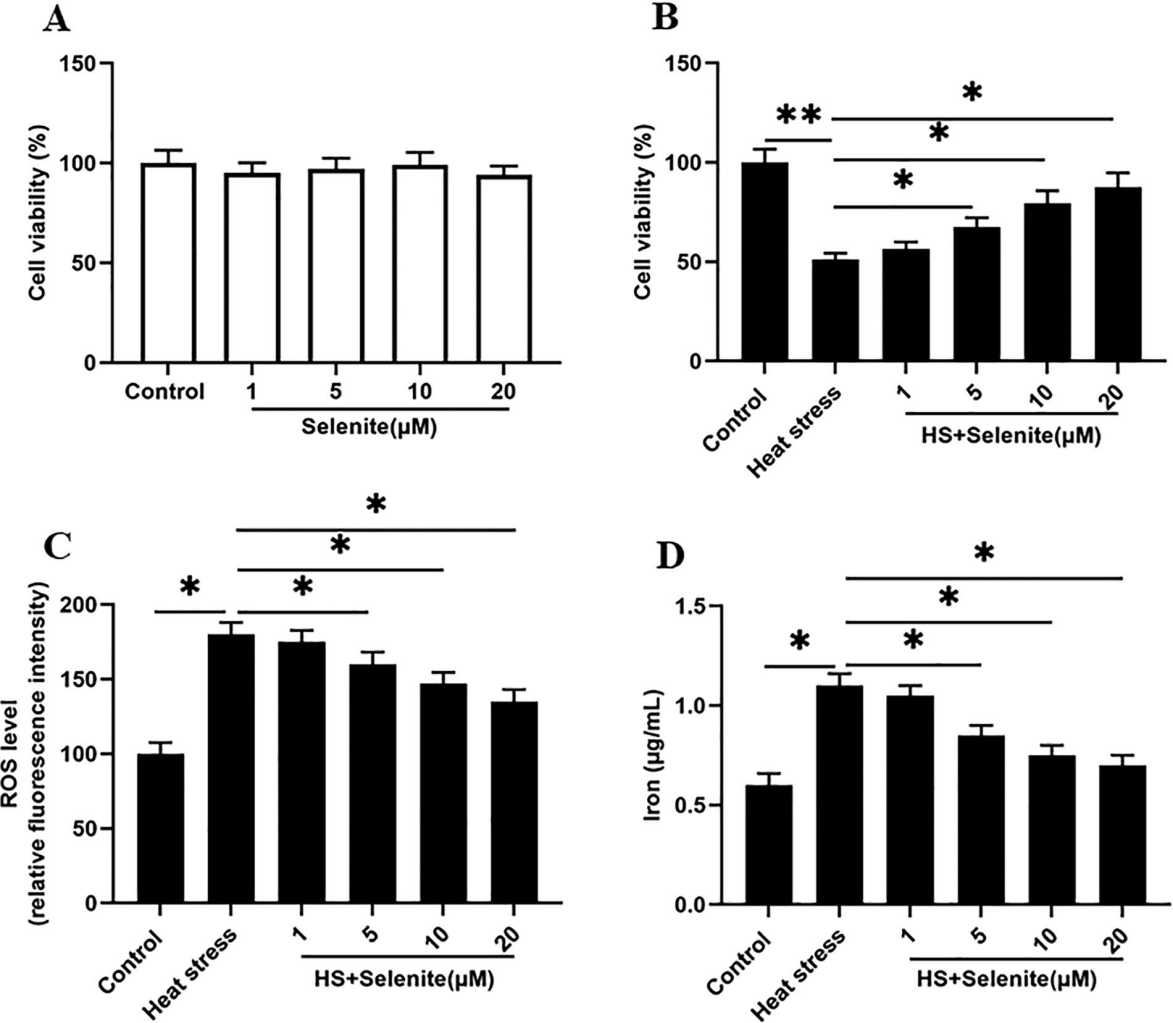
Selenium incubation reduces heat stress-induced ferroptosis-like death of GMECs. After adding a certain concentration of selenite (1, 5, 10, 20 μM) to heat-stressed GMECs for 24 h, the cell viability, iron ion concentration and ROS level were measured: (A) The negative control group was incubated with different concentrations of selenium to test the effect of selenium addition on cell viability. (B) MTT assay was used to detect the cell viability. (C) Determination of ROS content. (D) Determination of iron ion concentration by spectrophotometer. *P < 0.05 versus control, **P < 0.01 versus control, n=6.

We first incubated GMECs with selenium at a concentration of 1, 5, 10, 20 μM, and found that different concentrations of selenium had no effect on cell viability (Figure 2(A)). To investigate the effect of selenium on heat stress-induced GMEC ferroptosis-like death, heat stress-induced GMECs were incubated with different concentrations of selenium. We found that the cell viability was increased at a dose-dependent manner (Figure 2(B)). Simultaneously, selenium incubation significantly reduced the ROS accumulation and iron ion concentration of cells under heat stress, and all the responses were significant and positive related in a dose-dependent manner (Figure 2(C,D)).

### Selenium incubation improves GPX4 expression and SOD activity under heat stress

**Figure 3.**
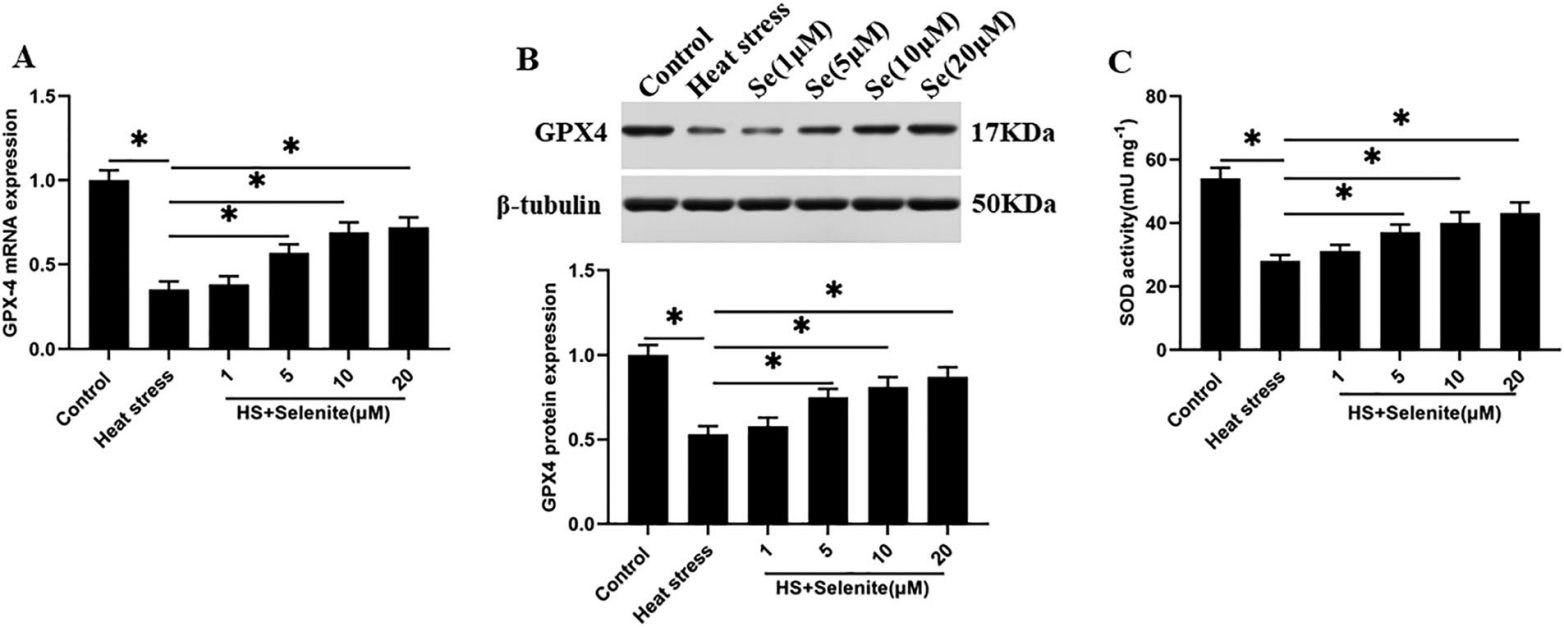
Selenium incubation improves GPX4 expression and SOD activity under heat stress. After adding a certain concentration of selenite (1, 5, 10, 20 μM) to heat-stressed GMECs for 24 h, the expression of GPX4 and the activity of SOD were detected: (A) RT-PCR was used to detect the expression of GPX4 nucleic acid. (B) Western blotting was used to measure the expression of GPX4 protein. (C) SOD determination kit was used to determine the activity of SOD. *P < 0.05 versus control, **P < 0.01 versus control, n=6.

To explore the effect of selenium on the expression of GPX4 and SOD activity in GMECs under heat stress. Heat stress-induced GMECs were incubated with selenium at a concentration of 1, 5, 10, 20 μM, and GPX4 mRNA and protein levels and SOD activity were detected. The results found that selenium incubation increased the expression level of GPX4 and SOD activity in GMECs in a dose-dependent manner (Figure 3(A–C)).

### SOD activation reduces heat stress-induced ferroptosis-like death of goat mammary epithelial cells

**Figure 4.**
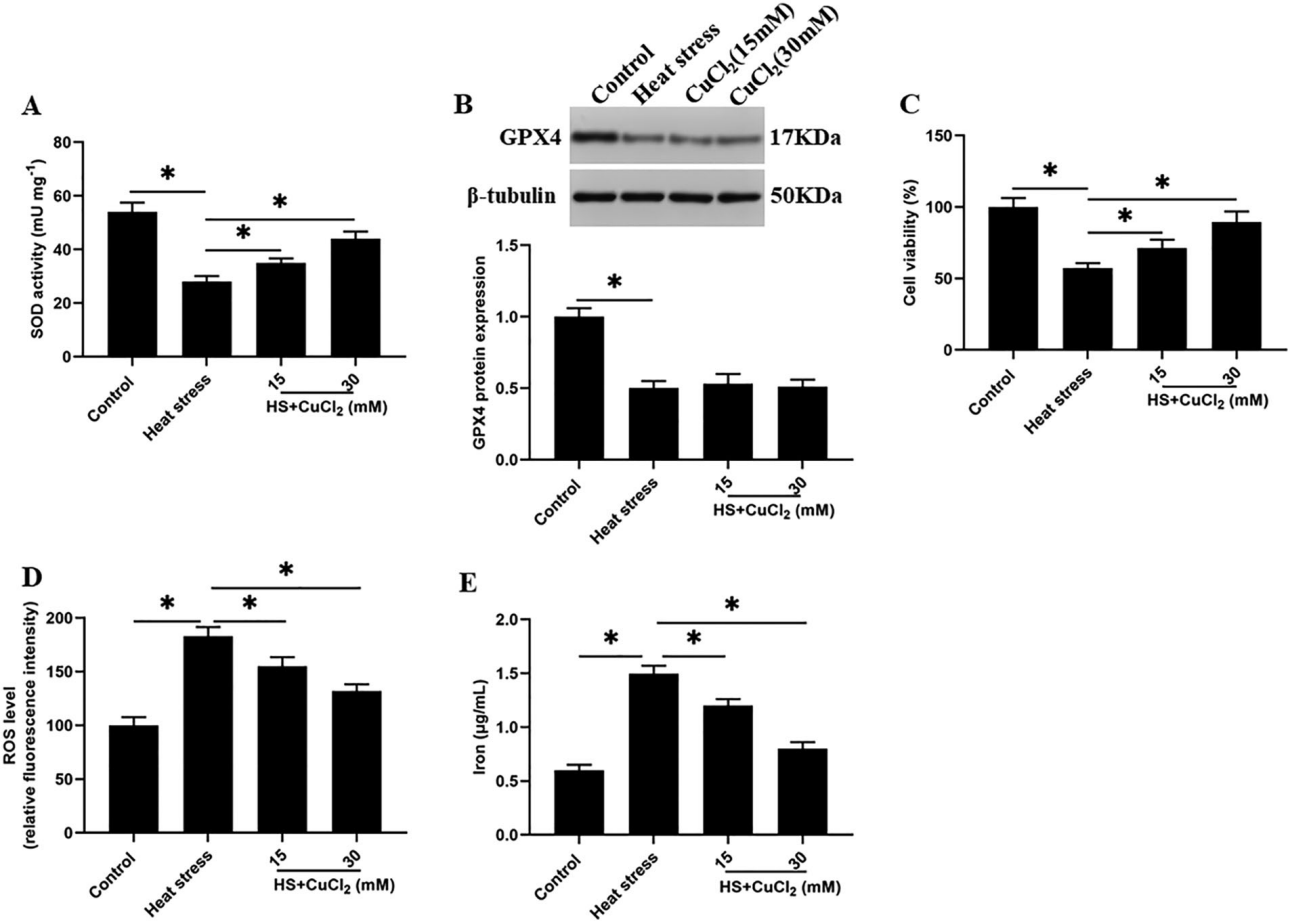
SOD activation reduces heat stress-induced ferroptosis-like death of GMECs. Adding a certain concentration of SOD activator CuCl_2_ (15, 30 mM) to heat-stressed GMECs. After 24 h of co-cultivation, the degree of cell ferroptosis-like death can be judged by detecting cell viability, iron ion concentration, and ROS level: (A) SOD determination kit was used to determine the activity of SOD. (B) Western blotting was used to measure the expression of GPX4 protein. (C) MTT assay was used to detect the cell viability. (D) Determination of ROS content. (E) Determination of iron ion concentration by spectrophotometer. *P < 0.05 versus control, n=6.

Previous studies reported that SOD could convert ROS to H_2_O_2_ to reduce the ROS accumulation (Burphan et al. 2018), while GPX4 could convert H_2_O_2_ to H_2_O (Schoeneberger et al. 2015). However, the role between SOD and GPX4 is still unclear. To investigate the interaction between them, we increased the activity of SOD by using a SOD activator CuCl_2_ under heat stress. Unexpectedly, after the SOD activity was increased, the cell viability was significantly increased, and the ROS accumulation and iron ion concentration were decreased in a dose-dependent manner (Figure 4(A,C,D,E)). However, the expression of GPX4 protein did not change significantly (Figure 4(B)).

### Knockdown of GPX4 inhibits SOD activity and increases ferroptosis-like death of GMECs

**Figure 5.**
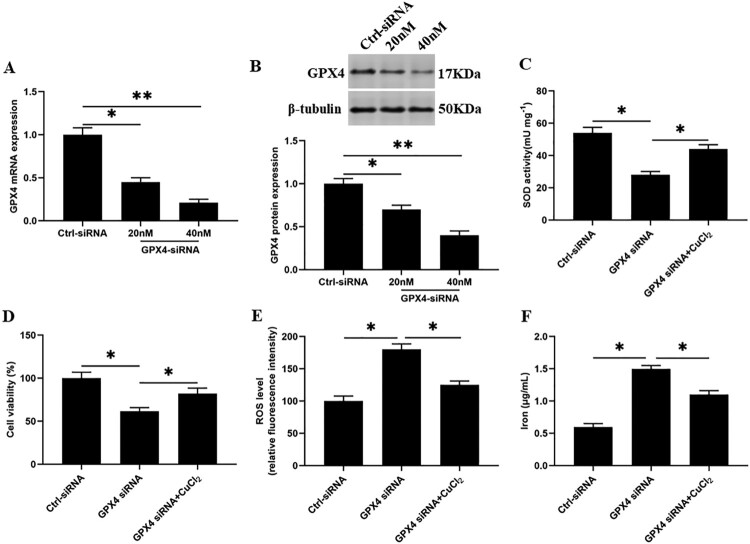
Knockdown of GPX4 inhibits SOD activity and increases ferroptosis-like death of GMECs. GMECs cultured in DMEM were treated by different concentrations of GPX4 siRNA (20, 40 nM), then cells and supernatants were isolated for functional determinations: (A) RT-PCR was used to determine the expression of GPX4 nucleic acid. (B) Western blotting was used to measure the expression of GPX4 protein. (C) GMECs were treated with 40 nM GPX4 siRNA and 15 mM CuCl_2_, cultured for 24 h, and then SOD determination kit was used to determine the activity of SOD. (D) MTT assay was used to detect the cell viability. (E) Determination of ROS content. (F) A spectrophotometer was used to determine the concentration of iron ions. *P < 0.05 versus control, n=6.

Next, we knocked down the GPX4 in GMECs, and the results showed that compared with the control group, the SOD activity and cell viability were significantly decreased (Figure 5(C and D)), and the accumulation of ROS and iron ion concentration were significantly increased (Figure 5(E and F)). After SOD activation, the SOD activity and cell viability in GMECs were significantly increased (Figure 5(C and D)), and the accumulation of ROS and iron ion concentration were significantly decreased compared with the GPX4 interference group (Figure 5(E and F)).

### Overexpression of GPX4 reduces SOD activity and reduces heat stress-induced ferroptosis-like death in goat mammary epithelial cells

**Figure 6.**
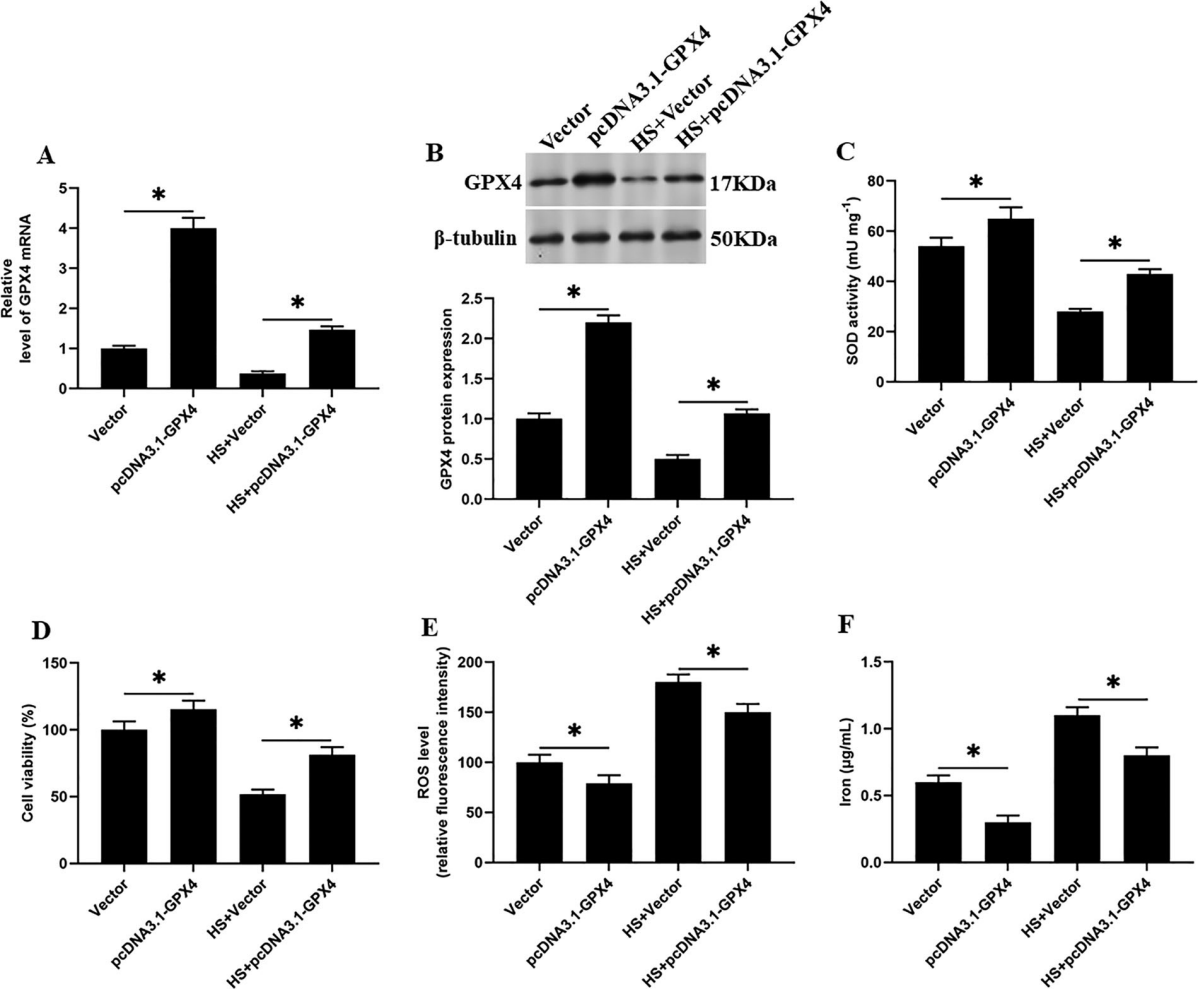
Overexpression of GPX4 reduces SOD activity and reduces heat stress-induced ferroptosis-like death in GMECs. The negative control group and heat stimulation group were transfected with 1 mg/mL adenovirus carrying GPX4, and then the cells and supernatant were separated for functional testing: (A) RT-PCR was used to determine the expression of GPX4 nucleic acid. (B) Western blotting was used to measure the expression of GPX4 protein. (C) SOD determination kit was used to determine the activity of SOD. (D) MTT assay was used to detect the cell viability. (E) Determination of ROS content. (F) A spectrophotometer was used to determine the concentration of iron ions. *P < 0.05 versus control, n=6.

We overexpressed GPX4 in heat stress-induced GMECs, and found that compared with the heat stress group, the SOD activity and cell viability were significantly increased (Figure 6(C and D)), and the accumulation of ROS and iron ion concentration were significantly decreased in GMECs (Figure 6(E and F)). Suggesting that GPX4 overexpression regulates the activity of SOD, which affects the ferroptosis-like death in GMECs induced by heat stress.

### mTOR signaling reversely regulates GPX4 expression and inhibits SOD activity under heat stress

**Figure 7.**
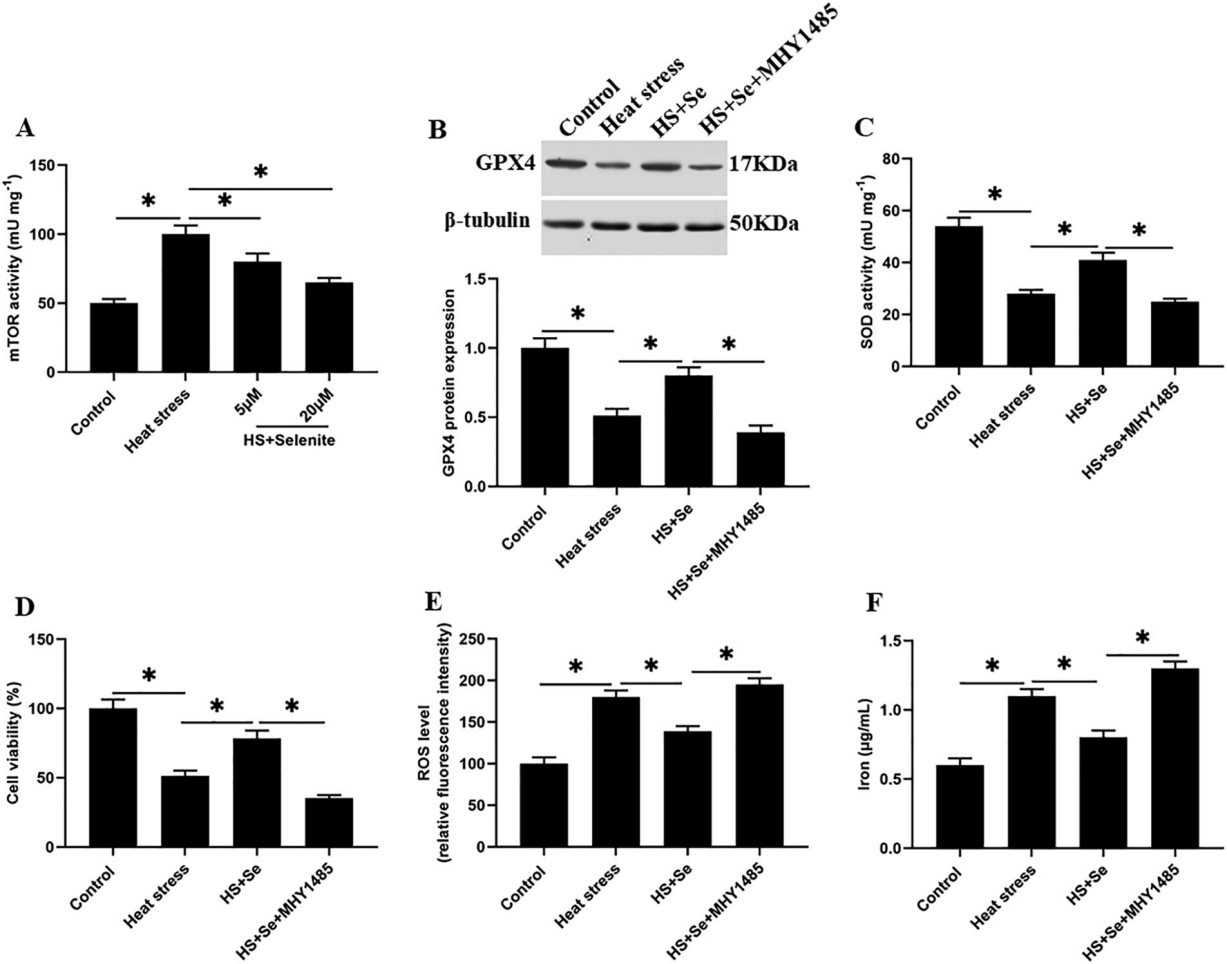
The activation of mTOR inhibits the expression of GPX4 and the activity of SOD. Adding 10 μM selenite or mTOR activator MHY1485 to heat-stressed GMECs. After 24 h of culture, the cells and supernatant were separated for functional determination: (A) mTOR determination kit was used to determine the activity of mTOR. (B) Western blotting was used to measure the expression of GPX4 protein. (C) SOD determination kit was used to determine the activity of SOD. (D) MTT assay was used to detect the cell viability. (E) Determination of ROS content. (F) A spectrophotometer was used to determine the concentration of iron ions. *P < 0.05 versus control, **P < 0.01 versus control, n=6.

We found that compared with the control group, the activity of mTOR (Figure 7(A)), the expression levels of phosphorylated mTOR (p-mTOR) (Supplementary Figure 1A and 1B), p-S6K1 and p-4EBP1 proteins in GMECs under heat stress were significantly increased (Supplementary Figure 1C-E). And compared with the heat stress group, selenium incubation decreased the activity of mTOR (Figure 7(A)) and the expression levels of p-mTOR (Supplementary Figure 1A and 1B), p-S6K1 and p-4EBP1 proteins in GMECs (Supplementary Figure 1C-E). To explore the effect of mTOR on heat stress-induced GMEC ferroptosis-like death, heat stress-induced GMECs were incubated with selenium or together with mTOR activator MHY1485, as well as incubated with mTOR inhibitor AY-22989, and then cell functions were detected. The results showed that compared with heat stress group, AY-22989 incubation significantly reduced the expression levels of p-mTOR (Supplementary Figure 1A and 1B), p-S6K1 and p-4EBP1 proteins (Supplementary Figure 1C-E), the accumulation of ROS and iron ion concentration (Supplementary Figure 1J and 1K), and increased the expression level of GPX4 protein (Supplementary Figure 1F and 1G), SOD activity and cell viability of GMECs (Supplementary Figure 1H and 1I). And compared with selenium treatment group, selenium combined with MHY1485 treatment significantly increased the expression levels of p-mTOR (Supplementary Figure 1A and 1B), p-S6K1 and p-4EBP1 proteins (Supplementary Figure 1C-E), the accumulation of ROS and iron ion concentration (Figure 7(E and F), Supplementary Figure 1J and 1K), and reduced the expression level of GPX4 protein (Figure 7(B), Supplementary Figure 1F and 1G), SOD activity and cell viability of GMECs (Figure 7(C and D), Supplementary Figure 1H and 1I). These results suggesting that selenium reduces the expression level of GPX4 and SOD activity by inhibiting the activation of mTOR signaling, and alleviates heat stress-induced ferroptosis.

## Discussion

Ferroptosis is a non-apoptotic cell death manner, which is characterized by iron dependence and ROS accumulation and changes in the morphology and function of mitochondria in the cell, finally, the cells are dead due to the accumulation of ROS (Park et al. 2019). Additionally, another signaling pathway is related to the inactivation of GPX4, resulting in ferroptosis (Xie et al. 2016). Currently, it is reported that the up-regulation of GPX4 expression could significantly inhibit ferroptosis-like death of Sertoli cells, and affect male reproductive ability to a certain extent (Li et al. 2018). In this study, we found that heat stress could increase the iron ion concentration and ROS accumulation in GMECs, and decrease the expression of GPX4 and the activity of SOD, which ultimately led to the ferroptosis-like death of GMECs. Simultaneously, a report found that selenocysteine utilization by GPX4 confers exquisite resistance to irreversible overoxidation (Ingold et al. 2018). Studies reported that diets containing selenium could promote the expression of GPX4 and the activity of SOD in poultry, and improve the production performance, meat quality and antioxidant capacity of poultry (Chen et al. 2017; Khan et al. 2018; Mengistu et al. 2021). Moreover, it is reported that adding selenium and vitamin E to the diet could increase the expression of GPX4 and the activity of SOD in the breast meat of broilers under summer heat stress conditions, eliminating ROS to a certain extent and stabilizing the antioxidant status of broilers in summer (Shahnawaz et al. 2018). Currently, the effect of selenium on goat mammary epithelial cells under heat stress is unclear. Our study found that selenium incubation could improve the antioxidant capacity by increasing the expression of GPX4 and SOD activity in GMECs under heat stress.

A study found that in the MI phase of mouse cardiomyocytes, the down-regulation of GPX4 and the decrease of SOD activity are related to the accumulation of intracellular ROS. Simultaneously, knockdown of GPX4 could induce the accumulation of lipid ROS at the early phase and then cause ferroptosis after a period of time (Park et al. 2019). It is reported that ROS accumulation is promoted following oxidative stress exposure to either H_2_O_2_ or erastin during GPX4 knockdown, and overexpression of GPX4 eliminates the oxidative stress, thereby protecting physiological processes in human pancreatic cancer cells (Peng et al. 2019). As the first line of defense for enzymes in the antioxidant system, SOD first converts cell damaged ROS into H_2_O_2_, and GPX4 plays a role in the downstream of SOD. In this study, we found that when SOD was activated under heat stress, the expression level of GPX4 protein was not affected. However, when we knocked down GPX4, the activity of SOD was significantly reduced. When we overexpressed GPX4 under heat stress, the activity of SOD was significantly increased, and the ferroptosis-like death of cells was alleviated. It is suggested that GPX4 promotes the activation of SOD.

Currently, most studies mentioned that the activation of mTOR could cause ferroptosis (Du et al. 2019; Li et al. 2019; Chen et al. 2020). Our study found that the mTOR signaling pathway was activated under heat stress. Moreover, studies reported that the activation of mTOR signal could inhibit the level of GPX4, which leads to ferroptosis (Han et al. 2020). It is reported that selenocysteine (ASC) could induce autophagy via the AMPK/mTOR pathway (Wu et al. 2015). Simultaneously, H_2_Se could interrupt the disulfide bond in HMGB1 and promote its secretion to inhibit the activation of mTOR signaling (Pan et al. 2019). A previous study showed that selenium nanoparticles (SeNPs) synthesized by *Lactobacillus casei* could reduce the phosphorylation level of mTOR protein, overproduction of ROS, adenosine triphosphate (ATP) level and mitochondrial membrane potential, alleviating hydrogen peroxide-induced intestinal epithelial barrier dysfunction (Yan et al. 2021). It is reported that after small interfering RNA target Selenoprotein U are transfected into chicken Sertoli cells, the mRNA and protein levels of the autophagy-related genes, including mTOR, PI3 K, and Akt are significantly reduced, compared with the control group, and cell apoptosis and autophagy are significantly increased (Sattar et al. 2018). These studies all illustrate a point that selenium may be involved in regulating the activity of mTOR, but the specific regulation mechanism is still unclear. In this study, we found that selenium incubation inhibited the activation of mTOR signaling, and reduced the accumulation of ROS and iron ion concentration, and increased the expression level of GPX4 protein, SOD activity and cell viability in heat stress-induced GMECs, and mTOR inhibitor AY-22989 had the same effect of selenium, while the addition of mTOR activator MHY1485 reversed the effects of selenium.

**Figure 8.**
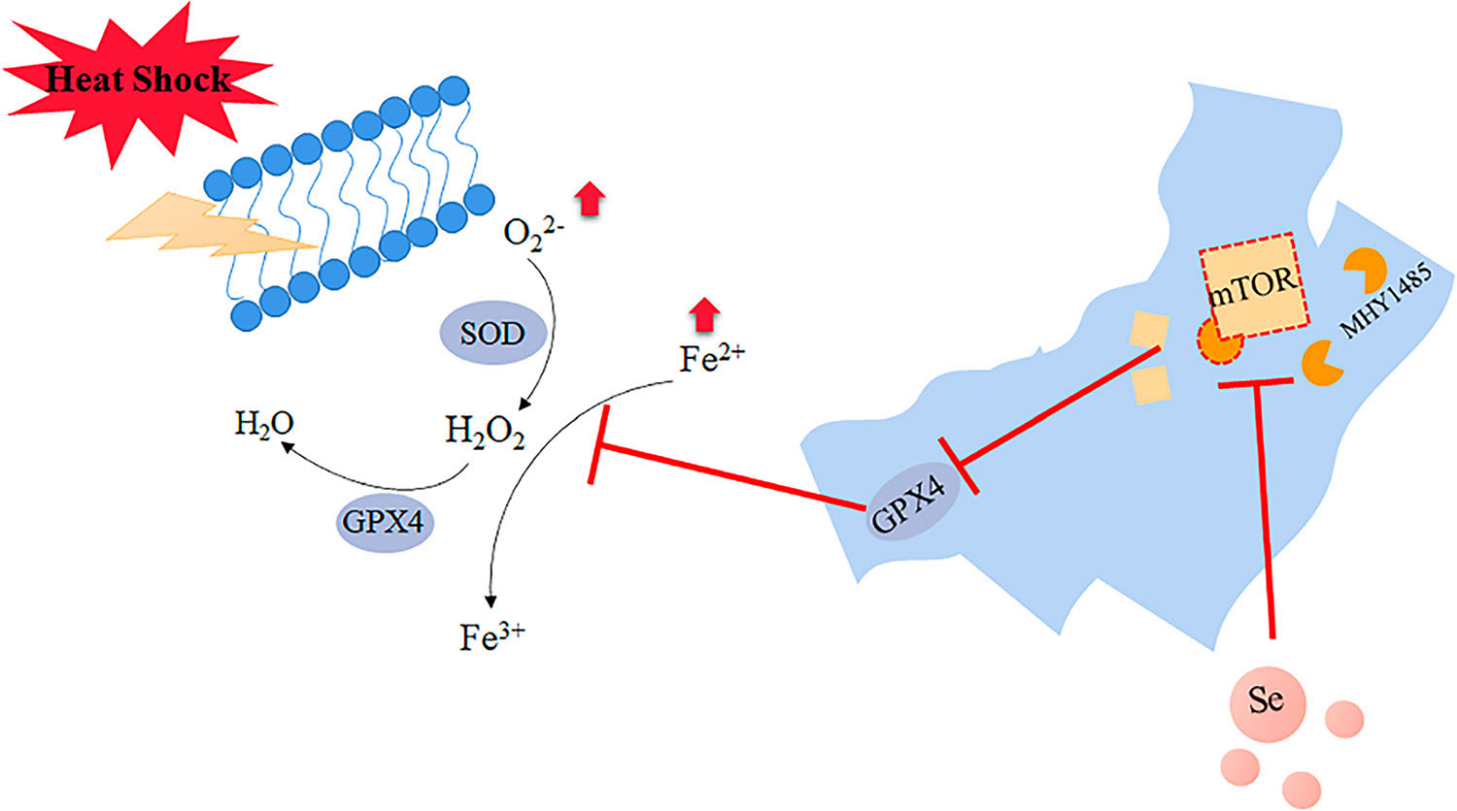
Selenium promotes the expression of GPX4 by inhibiting the activity of mTOR. Under heat stress treatment, the iron ion concentration and ROS level of GMECs increased significantly and caused ferroptosis-like death in the cells. The activity of mTOR was higher under heat stress, which further inhibited the expression of GPX4, the addition of selenium inhibited the activity of mTOR under heat stress and improved the expression of GPX4, and then the activity of SOD is increased, thereby improving ferroptosis-like death under heat stress.

In summary, our research found that selenium incubation could inhibit the activation of mTOR signaling pathway and increase the expression of GPX4 and SOD activity, thereby alleviating the heat stress-induced ferroptosis of GMECs (Figure 8), which provides a new idea for goats to resist the physiological reaction of mammary gland under heat stress.

## Supplementary Material

Supplemental Material

## Data Availability

All data generated or analyzed during this study are included in this published article.
