# Supplementary figures and images for: Se improves GPX4 expression and SOD activity to alleviate heat-stress-induced ferroptosis-like death in goat mammary epithelial cells
Source: Anim Cells Syst (Seoul). 2021 Oct 17;25(5):283–95. doi: 10.1080/19768354.2021.1988704 (PMC8567913; doi:10.1080/19768354.2021.1988704)

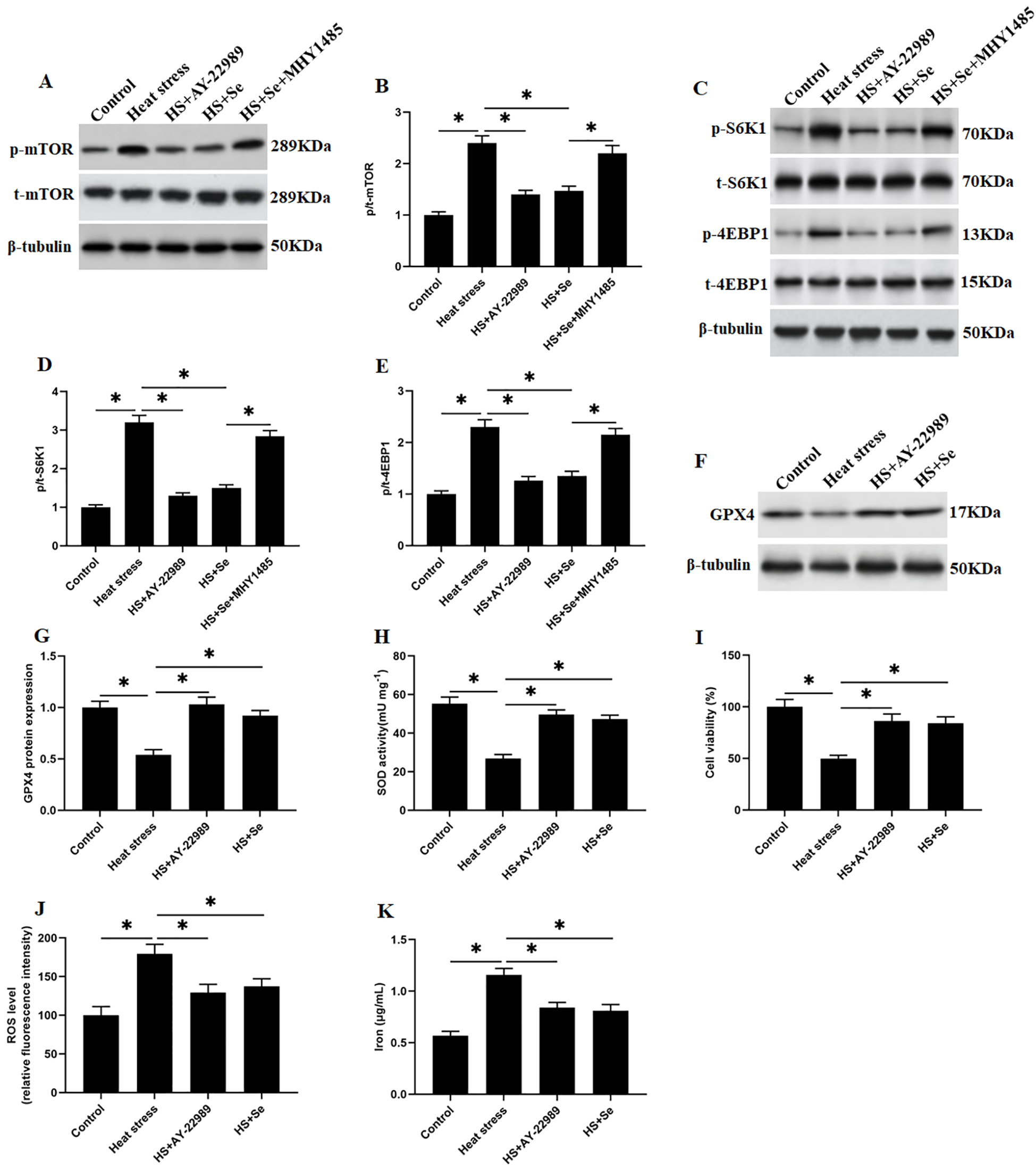

Supplement: Supplemental Material [file TACS_A_1988704_SM8571.tif]
